# The use of vanishing spray reduces the extent of rule violations in soccer

**DOI:** 10.1186/s40064-016-3274-2

**Published:** 2016-09-15

**Authors:** Otto Kolbinger, Daniel Link

**Affiliations:** Chair of Training Science and Sport Informatics, Technical University of Munich, Georg-Brauchle-Ring 60/62, 80992 Munich, Germany

**Keywords:** Vanishing spray, Evaluation research, Umpiring aids, Referees, Free kicks

## Abstract

**Background:**

More and more sport associations introduce innovative devices to support referees and umpires respectively, affecting a strong need for the evaluation of these devices. This study evaluates the use of the new vanishing spray for free kicks in the German Bundesliga. In more detail, the aim of the study is to investigate if the spray reduces violations of the required minimum distance and consequently the respective punishments, if it reduces errors concerning the distance set by the referee and if it leads to a higher success rate of free kicks.

**Methods:**

Therefore, 1833 free kicks of the 2013/2014 and 2014/2015 season of the German Bundesliga were screened using a self-designed observational system. For the statistical analysis two parallel samples were built of 299 free kicks each.

**Results:**

The results showed no decrease of free kicks with distance violations but a significantly lower extent of these violations (χ^2^ = 4.58; p < .05). However, none of these violations were punished appropriately. Concerning the success of free kicks, no significant impact was found neither for shots nor for crosses. In addition, no influence on the distance set by the referee could be identified.

**Conclusions:**

The main objective of the vanishing spray was basically realized, but the use didn’t lead to any further positive (side) effects. Due to the lack of punishment, the authors raise concerns about the current application of the minimum distance rule.

## Background

An increasing amount of innovative devices is introduced in several sports to support the umpires and referees respectively. In general these devices serve as another tool to ensure the legitimate outcome of sport competitions (Rock et al. 2013). The function of the tools is to compensate the limits of human perception that cause for example optical errors and flash lag effects (Oudejans et al. 2000; Helsen et al. 2006), as well as to eliminate bias that referees show towards the hosting team or players of their own race and the same country (Parsons et al. 2011; Pope and Pope 2015; Dohmen and Sauermann 2015). The devices can be divided up into three different groups. Devices that support the referees in their decision making, devices that replace the referees for certain decisions and devices that help them to enforce the rules of a sport. The new vanishing spray in soccer belongs to the latter category. Referees can use this spray to mark the required minimum distance of 9.15 m (10 yards) that players of the defending team have to obey before a free kick is taken. To mark the distance the referee draws a line between the ball and the goal that players of the defending team aren’t allow to cross until the ball is touched by the offensive team. Its use was finally approved at the 126th annual general meeting of the International Football Association Board (IFAB) in 2012, but the spray was already introduced in several competitions across South America since 2000. The vanishing spray attracted worldwide attention with its appearance at the FIFA World Cup 2014 and was introduced in several European competitions, like the German Bundesliga, before and during the 2014/2015 season.

Currently, there is still a lack of evaluation research for this device, representing a common issue in sports, concerning these kinds of devices. Especially the impacts of umpiring aids are neglected. In this connection, the impact is not only the achievement of the objectives but also other (side) effects of the innovation, which can be positive as well as negative. This is quiet surprising, as investigating the merit or worth of interventions, should be a main goal due to most of the common definitions of evaluation (Scriven 1991; Stufflebeam and Shinkfield 2007). The evaluations of the respective associations focus mostly, sometimes even exclusively, on technical parameters that the devices have to fulfill. This technical aspect has the advantage that it can be investigated under laboratory conditions and it represents the most obvious precondition for the introduction of a device. Thus, taking goal line technology as an example, the FIFA established a series of technical tests that the systems have to pass to get credited as an official device, mainly focused on the accuracy of the systems and real time detection. The same applies to scholarly studies (Psiuk et al. 2014; D’Orazio et al. 2009). The FIFA as well as other soccer associations didn’t collect data about costs and benefits of the goal line technology. Kolbinger, Linke, Link and Lames (2015) showed a very low incidence of scenes that could be resolved exclusively by goal line technology. They found less than four such incidences per season per league and therefore raised concerns about the cost-benefit ratio, especially considering the costs of round about 2.4 million per year. For the use of the Hawk-Eye technology in Tennis even the standard of the technical evaluations is questioned. In two articles considering the effect of the presentation of the technology on the publics’ understanding of science, Collins and Evans (2008, 2012) denounced the test-design of the International Tennis Federation. Nevertheless some studies investigated the use and the impact respectively of this device. Mather (2008) as well as Abramitzky et al. (2012) both found that slightly under 40 % of the challenges were successful, meaning that the technology didn’t confirm the umpires’ call. Abramitzky et al. (2012) also showed that it is only used for a very low share of points, almost exclusively for balls within 100 mm of the line (Mather 2008), but that successful challenges can increase the winning probability by up to over 25 %.

No evaluation research was run for the use of the vanishing spray in soccer yet, so the aim of this study is to overcome this lack by investigating five hypotheses. In addition to those, the using patterns of the device were described. The spray was introduced to help the referee to enforce the minimum distance rule and stemming discussions about the spot of the ball or wall respectively. Thus, the first hypothesis to be tested is that the spray leads to fewer violations (H 1.1)—or respectively a lower extent of violations (H 1.2)—of the required minimum distance by players of the defending team. According to the official laws of the game of the Deutscher Fußball Bund (DFB, trans. German Football Association), these violations should be punished with a yellow card and a repetition of the free kick. Taken this into account, it was checked if the spray affects fewer warnings and less free kicks that have to be retaken, to provide further information concerning the patterns of rule violations. Without reference of these targets, but as the innovation created more awareness of the required distance, a third hypothesis, that the spray reduces estimation errors of the set distance between the spot of the free kick and the wall, was investigated (H 2). In addition, we assumed that no violations of the required distance benefit the kicking team, resulting in a higher success rate for free kicks taken as crosses (H 3.1) as well as for those that were taken as shots (H 3.2).

## Methods

### Data recording

The data set consisted of all free kicks of the 2013/2014 and 2014/2015 season that were taken as shots or crosses with a distance less than 35.0 m of the goal line. By signing a contract of employment as a professional soccer player in the German Bundesliga, each players sings a statement of consent to being monitored during matches. The provided data included a match ID, the teams involved, event time, two dimensional coordinates of the spot of the free kick and whether it was taken as a shot or cross. Using a self-deigned observational system two specific trained experts collected data for further variables that are shown in Table 1. Therefore, specific videos of the free kicks were provided starting 90 s prior to the taking and ending 10 s after it.

**Table 1:** The collected variables inclusive their respective categories and definitions

Variable	Categories and definition
Use of vanishing spray	*True* or *false*
Local category	*Left/right near*: On the sides of the penalty box
	*Left/right far*: On the sides of the virtually extended penalty box between 16.5 and 35 m distance of the goal line
	*Central near left/right*: Inside the virtually extended penalty box with not more than 26.5 m distance of the goal line (penalty box plus 10 m). Left and right are divided by a virtual line in the middle of the field drawn at right angle to the goal line
	*Central far:* Otherwise, but within 35 m distance to the goal line
Players in wall	Numbers of defensive players in the wall
Violation of the minimum distance	*True:* At least one player passes the referees mark with his entire foot
	*False:* otherwise
Massive violation of the min distance	*True:* More than one player commits a *violation* or a player reduces the distance by more than 1 m
	*False: otherwise*
Punishment for violations	*Yellow card*: A yellow card is awarded
	*Verbal cautions*: The referee corrects the players verbally
	*None*: No punishment
Free kick retaken	*True* or *false*
Success of shots	*OnTarget:* A goal is scored, the ball hits the goals border or the goalkeeper makes a save
	*Missed:* Ball misses the goal or is blocked by a player outside the wall
	*Wall:* Ball is blocked by the wall
Success of crosses	*Successful:* A player of the offensive team is able to perform a shot or pass with the first touch after the cross
	*Not successful:* Otherwise

The distance between the spot of the ball and the wall was obtained with a custom made analysis software. Using homography, this software enables the user to determine points on the field by transforming video coordinates into real world coordinates. Therefore this software requires not just positional data of a match, but also the respective specific tracking video. These data and videos respectively were only available to the authors for a subsample for which the distance set by the referee could be obtained. The respective variable |DistanceError| shows the absolute difference to the regulatory 9.15 m.

### Reliability

The examination of the inter-rater-reliability showed excellent scores for most of the variables. Cohen’s Kappa reached a value of 1.00 for the use of the spray, .91 for the punishment of violations and 1.00 for the result of shots. The results of crosses and the identification of rule violations felt behind with .79 and .80 respectively but were still acceptable. The correlation coefficient (.98) as well as the relative observed agreement (92.6 %) for the numbers of players in the wall also showed a good agreement between the observers.

### Statistical analysis

To investigate the influence of the vanishing spray, sprayed free kicks after the introduction were compared to those before, based on the idea of evaluating interventions by comparing respective variables on different time points of a program (Cronbach 1963). Thus, the mentioned variables were collected for 1833 free kicks in total. 1108 of these free kicks were taken prior to the introduction of the vanishing spray and 725 after its introduction on the 8th day of the 2014/2015 season. As the free kicks after the introduction showed no consistency considering the use of the spray, it was decided to run a parallel study design to investigate the influence of the new device. The parallelization was performed in three steps. First, the free kicks were grouped into local categories on the basis of its two-dimensional coordinates (see Table 1). The number of players in the wall served as the second criteria, representing the perceived risk of the defending team. At last, the free kicks were paired in these categories on the basis of the shortest distance. Thus, two parallel samples were built with 299 free kicks each (N_Spray_/N_NoSpray_). 81 pairs of free kicks of these two groups represented the subsample for the investigation of the set distance by the referee.

The spatial distribution of the spray’s use was visualized using the ISOPAR method (Stöckl et al. 2011). Due to the different styles of the obtained variables different statistical analysis were run, after verifying the assumptions of normality. On the one hand, a paired *t* test was calculated for the set distance between the ball and the wall. On the other hand, Chi square tests for the violations, the punishment of these violations, retakes and the success of free kicks. All statistical analysis were performed with SPSS (Version 23.0; Armonk, NY; IBM Corp.), except the respective effect sizes Cohen’s d and Cramér’s V that were calculated manually. The magnitudes of the effect sizes were evaluated based on the limits: .10 (small), .30 (medium) and .50 (large) for Cramér’s V (Cramér 1946). The limits for Cohen’s d were .20 (small), .50 (medium) and .80 (large) (Cohen 1992).

## Results

**Fig. 1:**
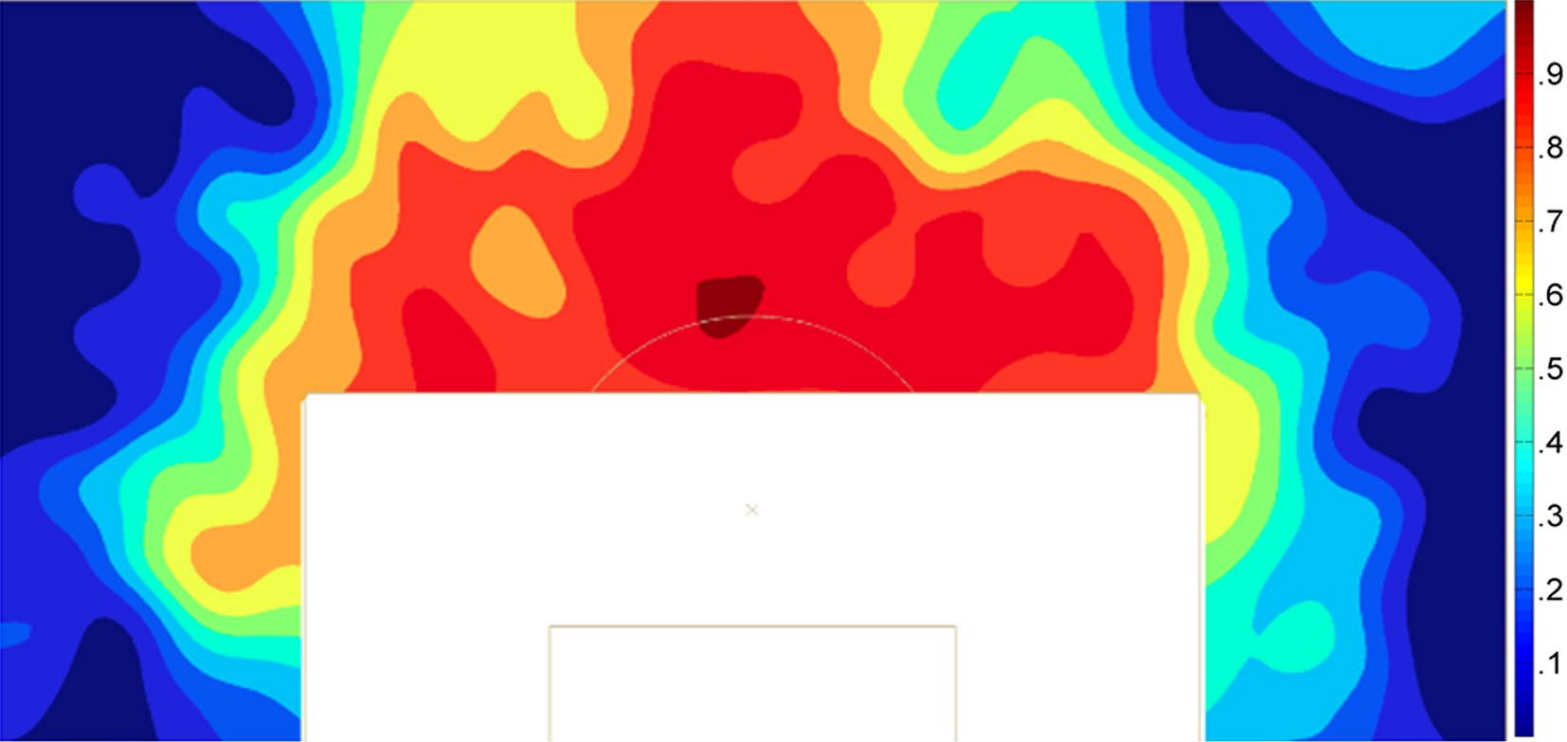
Spatial distribution of the proportion of sprayed free kicks (n = 725). *Dark red* illustrates a probability of 1.0 that the spray was used, *dark blue* a probability of .0

For 308 of the 725 investigated free kicks after the introduction the referees deiced to mark the regulatory distance with the vanishing spray. Figure 1 shows that the spray was used more likely for central free kicks, especially with decreasing goal distance. The spray was used for all the investigated respective free kicks with six or more players in the defensive wall and for 88.9 and 89.0 % for free kicks with walls of four and five players respectively. This number decreases further for free kicks with three (70.9 %), two (34.7 %) or one player (7.8 %) participating in the wall.

The introduction of the vanishing spray showed no significant influence on violations of the required minimum distance by players of the defending team, as the share of free kicks with violations remains on a similar level (OR = 1.02; 95 % CI .69–1.49). However, the share of massive violations decreases significantly by 6 %, representing a trivial effect size but an odds ratio of .60 (95 % CI .36–.99). Despite these violations of the required minimum distance, none of the free kicks of the sample was retaken and no yellow cards were awarded for this reason. Six verbal cautions for such violations were recorded for the treatment group, showing a significant increase with small effect size compared to the zero verbal cautions of the control group (χ^2^ = 6.06; p < .02; V = .10; OR 132.7; 95 % CI 1.12–8.56 × 10^3^).

The comparison of the set distance between the ball and the wall by the referee showed no influence of the vanishing spray in any direction. The average distance was 9.24 m for each group (SD_NoSpray_ = .87 m, SD_Spray_ = .93 m). The average absolute difference from 9.15 m, the prescribed distance by the laws of the game, showed a non-significant mean difference of −5.12 cm (CI −23.2 to 12.9). Overall, 50.6 % of the estimations are within a |DistanceError| of just .5 ms, 38.6 % showed an absolute error of .5 to 1.5 m and 10.8 % were off by more than 1.5 m.

The influence on the success of free kicks was evaluated separately for crosses and shots. For neither category significant influences could be identified. Despite the slight increase of goals from 8.6 to 9.3 % after the introduction, fewer shots were recorded in the “OnTarget” category (OR .91; 95 % CI .60–1.38) and more shots in the category “Missed” (OR 1.10; 95 % CI .74–1.65). The number of shots that were blocked by players standing in the wall decreased slightly by .3 % (OR .99; 95 % CI .63–1.54). The success of free kicks that were taken as crosses was just .65 as high after the introduction of the vanishing spray (95 % CI .29–1.46). As stated in Table 2, none of these changes were significant.

**Table 2:** Test statistics and effect sizes of the comparisons of the investigated variables before and after the introduction of the vanishing spray

Nominal variables	No spray	Spray	χ^2^	p	V
Violations (H 1.1)	25.4 %	25.8 %	.01	.925	.00
Massive violations (H 1.2)	16.7 %	10.7 %	4.58^a^	.032	.09
Successful crosses (H 3.1)	26.7 %	19.2 %	1.26	.261	.09
Success of shots (H 3.2)			.31	.857	.03
OnTarget	34.4 %	32.3 %	.23	.635	.02
Missed	38.8 %	41.2 %	.26	.611	.02
Wall	26.8 %	26.5 %	.00	.954	.00
Continuous variables			t		d
|DistanceError| (H 2)	.70 ± 0.64 m	.65 ± 0.58 m	.56	.577	.01

Continuous variables are stated as mean ± standard deviation

^a^Significant differences

## Discussion

The aim of this study was the evaluation of the new vanishing spray in soccer, which is used for 42.5 % of the respective free kicks, especially for those in promising positions. Therefore, five hypotheses were tested of which just one, the lower extent of rule violations, could be supported. The four other hypotheses concerning the amount of violations, the distance set by the referee and the success of either crosses or shots were not supported by the results. After separately discussing the findings regarding each hypothesis, the authors point out the significance of these findings and null-findings respectively for the evaluation research process itself as well as the understanding of the underlying phenomena.

A reduction in violations of the minimum distance, could not be proofed by the share of free kicks with violations. One out of four free kicks is still affected by such an incident. Nevertheless, the number of massive violation showed a significant decrease. Even though the respective effect size is just .09, the risk ratio shows a 1.56 times higher risk for massive violations for the control group. Thus, the spray had a positive effect on the extent of violations, and the main goal of the introduction was at least reached in curbing such incidences. In addition it is worthy to point out a limitation of this study. For sprayed free kicks, the observers could use the marked line as a reference, which (obviously) wasn’t possible for other free kicks. Consequently, for the latter category, the observers were instructed to note only violations if they can see the defenders moving towards the ball. Thus, if the wall reduced the distance while weren’t covered by the broadcast, this couldn’t be identified by the observers. This step was necessary to reach a good inter-rater agreement, but likely affected an underestimation of violations for free kicks without spray due to methodological reasons.

Despite this frequent incidence, the results also showed that violations of the distance rule were punished neither before nor after the introduction of the vanishing spray. Just a few verbal cautions were awarded after the introduction, but these verbal cautions not fulfilling the required extent of punishment. Two reasons could lead to this lack of punishment for rule violations. The first reason simply is that the referee can’t identify the violation. Since a free kick is a situation that has a comparatively clear structure in which the referees can focus more specific, the authors assume that the referees should be able to identify violations of the required minimum distance. In addition it should be even more possible for the referees with a marked line on the field, which now is available after introduction of the vanishing spray. Thus it is possible that some kind of unwritten rules come into play. According to D’Agostino (1995), unwritten rules can be seen as unofficial, implicit conventions that determine how the rules of a game are to be applied in specific circumstances. Translated for this situation, we suspect some kind of agreement between players of the different teams and the referee that the distance rule should be enforced less rigorously for free kicks. Considering that the correct punishment of such violations is even stated one more time in an extra annotation in the official laws of the game, this could be seen as a discrepancy between the protagonists on and off the field.

For positioning the wall the referees have to estimate the distance to the ball without any external aids. In more than 50 % of the free kicks the referees were within a distance of half a meter, but also a decent amount of errors of more than 1.5 meter occurred. We think the mean absolute error of .70 m before the introduction was still acceptable, especially considering that the mean distance to the ball is just 9 cm to high. The vanishing spray can’t support this process directly but led to more awareness of the distance in general. However, this didn’t affect the quality of estimation by the referees as there was no significant decrease in the absolute error.

Round about a third of the shots in this study were on goal, slightly more than one out of four is blocked by the wall and the remaining round about 40 % missed the target or were blocked by another player outside the wall. The results are pretty similar to previous findings of Carling, Williams and Reilly (2005). In an investigation of 152 attempts of the World Cup 2002 they found a rate of 8 % for goals and round about 35 % on goal (combining their respective categories). They state that only seven percent of the free kicks were blocked by the wall but a surprisingly high amount of free kicks that went through the wall (15 %), which sums up to a similar rate compared to this study.

The overall rate of successful free kicks taken as crosses was short to 23 %. To compare the values to those of previous studies, it is necessary to consider that we used a different definition of “successful”. Casal et al. (2014) for instance stated that of 21.8 % of indirect free kicks resulted in a shot in international competitions. As mentioned above in the current study a cross was rated successful if the ball reached a player of the same team in a way that this player could execute a controlled action with the ball. That’s the only goal that the player who is taken the free kick can control and vice versa for what he has to pass the defensive wall. Thus, this definition of “successful” is more appropriate for this study and arguably also for other studies that focus rather on the taking player.

The use of vanishing spray should benefit the offensive team. Despite a slight increase of the goal rate, this could not be proved with the results of this study. No significant differences were found for the success of free kicks, neither for free kicks taken as shots nor for crosses. The rate of successful crosses even decreased by 7.5 % which, however, wasn’t a significant difference. Comparing the free kicks with massive violations of the distance rule indicate that this violations don’t affect the outcome of the free kick, but there wasn’t a large enough sample to run reliable analysis. Another reason could be that the curbing of rule violations is too small to create a significant benefit for the kicking team, especially considering the variations in the distance set by the referee.

Summed up, the introduction of the vanishing spray basically fulfilled its main goal, by reducing the extent of violations of the minimum distance rule, but didn’t lead to further positive effects. Especially for the purpose of evaluation research, the respective null findings are as valuable as other findings to estimate the worth of an intervention. Rather spoken, this worth is estimated by the respective stakeholders, which must thoroughly consider all results of an evaluation for the decision making process (e.g. Stufflebeam 1983). This study illustrates an interesting example, as more and more competitions start to use the vanishing spray, despite an effect that seems to be rather small. Thus, in the eyes of the majority of the respective stakeholders the merit seems to be big enough to outweigh the disadvantages. A similar case was already made for another device in soccer before, the goal line technology (see Kolbinger et al. 2015).

In addition, evaluation researches create new knowledge about the underlying phenomena (Stufflebeam and Shinkfield 2007), which is especially true in this study for the distance set by the referees or the application of the respective set of rules. For the first time, it was shown that there is a discrepancy between the official set of rules for free kicks and its execution on the field, which is also true for the investigation of the referee’s estimation of the minimum distance. The respective findings both raise questions concerning the use of the vanishing spray. Users of the device need to be aware that the device controls the compliance of a certain distance, which is in fact not the intended distance for most of the time (Oldfather and Fernholz (2009) describe a similar phenomenon concerning the first down marks in American Football). The respective associations also need to be aware, that there is a lack of punishment for minimum distance rule violations, which can’t be solved solely by the use of vanishing spray.

## Conclusion

The findings of this study point out the importance of evaluating innovative devices that support the referees in game sports. Based on the objective targets of the respective association the authors showed that the vanishing spray fulfilled its main goal by decreasing the extent of violations at least in some extent. But in addition to that, these evaluations not just generate feedback concerning the new device but also on the underlying phenomena. In this study, the results also indicate a lack of application for the distance rule. Despite a frequent incidence of violations of the minimum distance, none of the investigated free kicks was retaken neither a yellow card was awarded due to this reason. The authors suggest two ways to overcome this discrepancy. On the one hand, the associations could try to increase the awareness of the appropriate punishment of violations of the distance rule. On the other hand, the rule itself could be adapted by changing the extent for the punishment.
